# A Good Appointment

**Published:** 1886-01

**Authors:** 


### A GOOD APPOINTMENT.

We are much pleased to announce the appointment of our talented young friend Dr. Frank A. Maxwell, of Delvalle,to be Assistant Physician to the State Lunatic Asylum, vice Dr. Johnson, married and resigned. Dr. Johnson will locate in Austin. Welcome, Doctor.

